# The role of tumor-associated macrophages in oral squamous cell carcinoma

**DOI:** 10.3389/fphys.2022.959747

**Published:** 2022-08-29

**Authors:** Yiwen Xue, Xiao Song, Siyu Fan, Runzhi Deng

**Affiliations:** ^1^ Department of Oral and Maxillofacial Surgery, Nanjing Stomatological Hospital, Medical School of Nanjing University, Nanjing, Jiangsu, China; ^2^ Central Laboratory of Stomatology, Nanjing Stomatological Hospital, Medical School of Nanjing University, Nanjing, Jiangsu, China

**Keywords:** TAMs, OSCC, invasion and metastasis, angiogenesis, immunosuppression

## Abstract

Oral squamous cell carcinoma (OSCC) is a common head and neck cancer with a high recurrence rate and a low 5-year survival rate. Tumor-associated macrophages (TAMs) are important immune cells in the tumor microenvironment, which play an important role in the progression of many tumors. This article reviews the origin, and the role of TAMs in the invasion, metastasis, angiogenesis and immunosuppression of OSCC. Therapeutic strategies targeting TAMs are also discussed in hopes of providing new ideas for the treatment of OSCC.

## 1 Introduction

The incidence of head and neck cancer ranks sixth among all cancers (Sun S. et al., 2018; Jin et al., 2019), of which oral cancer is an important head and neck cancer (Dong et al., 2017; Park et al., 2019). Approximately 377,000 patients were newly diagnosed with oral cancer and 178,000 deaths were caused by this cancer globally in 2020 (Sung et al., 2021). From these statistics, oral squamous cell carcinoma (OSCC) accounted for 90% of all oral cancers (Wang et al., 2020). Surgery is currently the preferred treatment for most OSCC, and sometimes this is accompanied by radiotherapy, chemotherapy or immunotherapy (Zanoni et al., 2019). Although great progress has been made in the diagnosis and treatment of OSCC, the prognosis of OSCC has not improved significantly over the past few decades, with the recurrence rate still being high and the 5-year survival rate still remaining low (Li Z. et al., 2020; Yang Z. et al., 2021).

Tumors are not only masses of proliferating tumor cells, but are complex tissues composed of many different cell types, which together constitute the tumor microenvironment (TME), forming a complex network supporting tumor progression (Hanahan, 2022). The OSCC TME consists of a variety of cell types, including local stromal cells, such as fibroblasts, and distant recruitment cells, including endothelial and, immune cells (Kalogirou et al., 2021). There are various innate (macrophages, dendritic cells, lymphocytes, and natural-killer [NK] cells) and adaptive (T and B cells) immune cells in the TME, among which CD8+T cells, NKs, and DCs mainly play an anti-tumor role (Guillerey, 2020). Tumor-associated macrophages (TAMs) are important immune-effector cells in the TME, which are closely related to the occurrence, development and prognosis of various solid tumors.

## 2 The origin, classification, and function of tumor-associated macrophages

The occurrence of OSCC is accompanied by a change in the immune microenvironment. In normal oral mucosa, the proportion of macrophages is less than 20%, however, in hyperplasia and cancer tissues, the infiltration of macrophages is significantly increased (Rangel et al., 2022). To study the dynamic changes in the immune environment during the development of oral carcinoma, the transplanted tumor model in 4-NQO mice was studied. The results showed that during the progression of normal mucosa to OSCC, in addition to the change of CD3+T cell infiltration, the proportion of M2 macrophages increased significantly (Bouaoud et al., 2021). CD68, CD80, and CD163 are commonly used macrophage-surface markers. CD68 is a general-macrophage marker, whereas CD80 and CD163 are markers for M1 and M2 macrophages, respectively (Mantovani et al., 2002; Weber et al., 2015). Immunohistochemical staining of CD68, CD80, and CD163 on the normal oral mucosa and oral leukoplakia revealed that the three macrophage markers were rarely found in the normal oral mucosa. Whereas, the abundance of CD163 in tissues with moderate leukoplakia was significantly higher compared to those without dysplasia (Mori et al., 2015). An immunohistochemical study was performed on 201 tissue samples, including 50 malignant leukoplakia, 53 non-malignant leukoplakia, 49 OSCC corresponding to malignant leukoplakia, and 49 normal oral mucosae samples. In this study, the abundance of CD68 and CD163 in leukoplakia with malignant transformation were higher than those in leukoplakia without malignant transformation, while there was no significant difference in CD68^+^ macrophage density between leukoplakia without malignant transformation and normal oral mucosa tissue samples. The abundance of CD68 and CD163 in OSCC were significantly higher than those in normal oral mucosa and oral leukoplakia (Weber et al., 2020). The above studies indicate that the phenotype and number of macrophages in the cancerous area changes as oral cancer progress.

The majority of macrophages in the TME are bone marrow-derived, and enter tumor tissues through peripheral blood, and are then called tumor-associated macrophages (TAMs) (Vitale et al., 2019). In the OSCC TME, TAMs are dynamic and are a collection of a wide range of macrophage subtypes. At the early stage of tumor progression, TAMs mainly present the M1 phenotype, with anti-tumor properties (Boutilier and Elsawa, 2021). M1 TAMs have a high antigen-presentation ability, can promote the proliferation of CD8+T and NK cells through IL-6, IL-12, and TNF, and enhance their cytotoxicity to induce tumor cells apoptosis (Umemura et al., 2020). Tumor cells can secrete CSF-1, which binds to the CSF-1R receptor on the surface of TAMs to promote their polarization to M2 TAMs. The simultaneous activation of hypoxia-inducible factor-1 (HIF-1) and hypoxia-inducible factor-2 (HIF-2) in the TME and NF-Kβ signaling pathway can also lead to the M2 polarization of macrophages (Dan et al., 2020; Ai et al., 2021). M2 TAMs promote tumor growth, metastasis, and angiogenesis, while also enhancing tumor immune escape (Mori et al., 2011). In most solid tumors, M2 TAMs presence is negatively associated with patient prognosis. Recent studies have shown that, in addition to bone marrow-derived monocytes, tissue-resident macrophages are also the source of TAMs in different tumor types. For instance, in brain glioma and pancreatic ductal carcinoma, studies have shown that both tissue-resident macrophages and peripheral blood mononuclear cells constitute the TAMs population and promote tumor progression (Bowman et al., 2016; Zhu et al., 2017). Similarly, in OSCC, TAMs may not only originate from monocytes, but tissue-resident macrophages may also constitute a source of TAMs. However, there is still no direct evidence that tissue-resident macrophages are a component of TAMs in OSCC, which is a topic that warrants further studies.

Troiano et al. showed that, in head and neck squamous cell carcinoma, the presence of CD163+TAMs was significantly negatively correlated with the prognosis of patients (Troiano et al., 2019). Other studies have shown that, in head and neck squamous cell carcinoma, TAMs mainly exhibited the M2 phenotype (Chiu et al., 2015; Weber et al., 2016; Ai et al., 2021). Mori et al. performed an immunohistochemical study on OSCC specimens and found that M2 macrophages accounted for a large proportion of infiltrated TAMs in OSCC, and that the number of M2 macrophages was positively correlated with the pathological stage of OSCC (Mori et al., 2011). Chaudhari et al. found that the number of M2 macrophages was significantly positively correlated with tumor stage and lymph node metastasis in OSCC, indicating that the number of M2 macrophages in the TME was related to the OSCC progression (Chaudhari et al., 2020). Taken together, the abovementioned studies (and additional ones, all summarized in Table1) investigating the role of TAMs in OSCC suggest a critical contribution of TAMs to OSCC progression.

**TABLE 1:** Role of TAMs in OSCC.

Year	Samples	Markers	Mouse/Human	Role of TAMs in OSCC	References
2020	125	CD68/CD163	Human	promote the immune escape of OSCC	Guo et al. (2020)
2016	127	CD163	Human	promote the progression of OSCC	Haque et al. (2019)
2011	50	CD163	Human	malignant transformation of OSCC	Hu et al. (2016)
2019	44	CD163/CD204/CD206	Human	promote the invasion	Kubota et al. (2017)
				and metastasis of OSCC	
2017	46	CD163/CD204	Human	promote	Li et al. (2019)
				immunosuppression of OSCC	
2020	32	CD206/CSF-1	Mouse	Promote the invasion and metastasis of OSCC	Mori et al. (2011)
2017	70	CD68/CD163/CD204	Human	promote lymphangiogenesis and lymph node metastasis of OSCC	Yamagata et al. (2017)
2019	173	CD163	Human	promote angiogenesis of OSCC	Suarez-Sanchez et al. (2020)
2016	11	CD11b/CD206	Human	Promote OSCC angiogenesis, leading to recurrence after radiotherapy	Okubo et al. (2016)
2018	72	CD68	Human	promote angiogenesis of OSCC	Sun et al. (2018a)

## 3 Tumor-associated macrophages promote oral squamous cell carcinoma invasion and metastasis

Local invasion and distant metastasis are hallmarks of tissue malignant transformation (Hanahan and Weinberg, 2011b). OSCC is a highly aggressive squamous cell carcinoma with local lymph node and distant metastasis (Leemans et al., 2011). Epithelial-mesenchymal transition (EMT) enables transformed epithelial cells to acquire the abilities of invasion, metastasis and anti-apoptosis (Zhao et al., 2017; Yang C. et al., 2021). EMT-induced transcription factors can coordinate invasion and metastasis (Su et al., 2014). Loss of E-cadherin and increased the abundance of the mesenchymal marker vimentin are two characteristics of EMT (Hong et al., 2015).

Tumor cells at the leading edge of tumor progression can establish a microenvironment suitable for tumor progression after undergoing EMT (Moustakas and Heldin, 2007). Macrophages are recruited to this edge by tumor-derived chemotactic agents and are a major infiltrating cell type in the leading edge (Condeelis and Pollard, 2006). After reaching the leading edge of tumor progression, TAMs can promote tumor cells motility and hydrolytic remodeling of the extracellular matrix (ECM) by secreting epidermal growth factors (EGFs) and other pro-migration factors and regulating the production of collagen fibers to enhance tumor cells invasion (O'Sullivan et al., 1993; Singh et al., 2010). In human OSCC, TAMs can induce EMT in tumor cells, thus enhancing the invasion and metastasis of OSCC (Hu et al., 2016). Simultaneously, with the increase in TAMs number, the presence of E-cadherin decreased significantly in OSCC. After treatment with TAMs-conditional medium, OSCC cells exhibited a fibroblast-like appearance, together with decreased E-mucin and increased-vimentin protein levels. This suggested that TAMs could promote the EMT of OSCC, enhancing the invasion and metastatic capacity of OSCC (Fan et al., 2014; Hu et al., 2016; Alves et al., 2021). CSF-1 stimulates the survival, proliferation, and differentiation of monocytes and promotes the proliferation and motility of macrophages (Goswami and S., 2005). In the TME, tumor cells secrete CSF-1, which bind to the macrophage CSF-1R receptors to promote their transformation into TAMs (Achkova and Maher, 2016). Studies using mouse-xenograft models have shown that when CSF-1 signaling was blocked, TAMs number in the tumor area was significantly reduced, and tumor growth was inhibited, indicating that blocking CSF-1 signaling can attenuate tumor invasion and metastasis (Li M. et al., 2020). EGF is a chemokine in the TME, which can accelerate tumor metastasis by promoting the migration and invasion of tumor cells (Du et al., 2013; Yin et al., 2019). Immunofluorescence showed that TAMs in OSCC expressed EGF, and flow-cytometry analysis showed that TAMs produced EGF. This suggested that TAMs could promote the invasion and metastasis of OSCC by secreting EGF in the TME (Haque et al., 2019). Yamagata et al. showed that CD163+ macrophages could promote lymphangiogenesis by expressing VEGF-C and contributed to regional lymph node metastasis in OSCC (Yamagata et al., 2017).

Taken together, EMT is characteristic of OSCC invasion and metastasis. In OSCC, TAMs can simultaneously reduce the presence of E-cadherin, and induce extensive EMT by secreting bioactive substances, promoting the invasion and metastasis of OSCC. However, tumor invasion and metastasis are complex process, and the mechanism of TAMs promoting OSCC invasion and metastasis remains unclear.

## 4 Tumor-associated macrophages promote oral squamous cell carcinoma angiogenesis

Similar to normal tissues, tumor growth requires nutrition and oxygen. Angiogenesis, one of the hallmarks of cancer, plays an important role in tumor progression (Hanahan and Weinberg, 2011a). In 1971, Judah Folkman first proposed that tumor growth depends on angiogenesis, the formation of new blood vessels in tumor tissue that provide nutrients and oxygen to the tumor while discharging metabolic waste and carbon dioxide (Okubo et al., 2016). In the TME, TAMs participate in tumor angiogenesis by releasing growth factors such as CCL2, CXCL8, CXCL12, EGF, IL-1β, IL-8, PIGF, TGF-β, and VEGF-A (Wyckoff, 2004; Chen et al., 2011; Tang et al., 2017; Yang et al., 2019).

The microvessel density (MVD) and the number of macrophages in the OSCC tumor area were significantly higher than those in the tumor-free margin and normal mucosa. The number of macrophages was positively correlated with MVD, indicating that TAMs were associated with angiogenesis in OSCC (Valverde Lde et al., 2016). Further studies have found that TAMs can promote angiogenesis in OSCC by activating the Hedgehog (Hh) signaling pathway (Petty et al., 2019). TGF-β plays an important role in tumor progression and metastasis (Costa et al., 2013). Multiple studies have shown that the TGF-β/Smad3 signaling pathway is associated with tumor progression and poor prognosis. Sun et al. showed that TGF-β can induce TAMs to secrete more VEGF by activating the TβRII/Smad3 signaling pathway in OSCC, promoting angiogenesis (Sun H. et al., 2018). PFKFB3 is a key enzyme in the glycolysis of endothelial cells and can mediate tumor progression. The expression of PFKFB3 in OSCC is positively correlated with the amount of M2 macrophages and MVD, suggesting that PFKFB3 may promote angiogenesis OSCC indirectly by modulating TAM numbers within the primary tumor (Li et al., 2019).

Current research shows that TAMs number is positively correlated with MVD in OSCC, and can promote angiogenesis in OSCC by secreting bioactive substances and activating signaling pathways. Therefore TAMs play an important role in OSCC progression.

## 5 Tumor-associated macrophages promote the immunosuppression during the progression of oral squamous cell carcinoma

Angiogenesis and vasculature remodeling within the malignantly transformed tissue causally influences the recruitment and infiltration of additional immune cells (Okubo et al., 2016). Within the tumor immune microenvironment, TAMs are known to exert immunosuppressive functions by promoting regulatory-T cell conversion within the primary tumor, and immunosuppression has been recognized as a hallmark of tumor progression (Hanahan and Weinberg, 2011b; Kalogirou et al., 2021; Kos et al., 2022).

In oral precancerous lesions, such as leukoplakia, M2 macrophages have been reported to create an immunosuppressive microenvironment. Immunohistochemical analysis of resected oral leukoplakia specimens showed that, compared with patients that exhibited low CD163+ macrophage amounts, leukoplakia lesions exhibited a high amount of CD163+ macrophages, which was usually accompanied by abnormal Ki-67 expression and keratin loss (Bouaoud et al., 2021). Meanwhile, *in vitro*, M2-macrophages conditioned medium was able to induce the production of immunosuppressive IL-10 in human oral epithelial cells *in vitro*. IL-10 levels were positively correlated with FOXP3+regulatory-T cells (Shigeoka et al., 2019).

TAMs promote tumor progression by shaping the tumor-immunosuppressive microenvironment. Within OSCC, TAMs secrete inhibitory factors, such as IL-10, TGF-β, and PEG2, to inhibit the function of CD8+T and NK cells (Mantovani et al., 2002; Gasparoto et al., 2010; Costa et al., 2013; Lyford-Pike et al., 2013; Wang et al., 2018). In non-small cell lung cancer, melanoma, head and neck squamous cell carcinoma, stomach cancer, and other cancers, TAMs overexpress PD-L1 and CTLA-4, which leads to inhibition of the TCR-signaling pathway and promotes the formation of a tumor immunosuppressive microenvironment (Egen et al., 2002; Hartley et al., 2018). Faustino et al. showed that, in OSCC, the presence of CD68^+^ and CD163+TAMs is associated with the high PD-L1 levels, which binds to the PD-1 receptor on the surface of activated-T cells and transmits inhibitory signals to T cells.This prevents the immune system from attacking tumor cells and enables these cells to obtain the ability to escape the immune system (Schmid et al., 2018; Kelly et al., 2019.; Suarez-Sanchez et al., 2020). Jiang et al. found that, compared with macrophages in the surrounding tissues, TAMs with high PD-L1 levels within the OSCC microenvironment could induce apoptosis of T cells. Peripheral blood-derived mononuclear cells (PBMCs) showed low abundance of PD-L1, however, after co-culture with the conditional medium from OSCC cells, the levels of PD-L1 were significantly increased, consequently promoting T-cell apoptosis. This indicated that, in OSCC, PBMCs were polarized into M2 macrophages in the TME, achieving high PD-L1 levels, which further played an immunosuppressive role in the progression of OSCC (Jiang et al., 2017). Immunofluorescence staining of OSCC-tissue samples revealed TAMs to have high protein amounts of IL-10 and PD-L1. TAMs were shown to induce the apoptosis of T cells through PDL-1 and IL-10 production, suggesting that TAMs may mediate the immune escape of OSCC cells, therefore ultimately promoting the invasion and metastasis of OSCC (Kubota et al., 2017).

Overall, these results suggest that M2 macrophages can create an immunosuppressive microenvironment within OSCC and oral-precancerous lesions, which protect tumor cells from immune-system mediated recognition and cell death.

## 6 Immunotherapy targeting tumor-associated macrophages

Numerous studies have shown that TAMs can promote tumor progression through various mechanisms in many solid tumors, and that TAMs number is significantly negatively correlated with patient prognosis (Nadella et al., 2020). Immunotherapy targeting TAMs has become a potential strategy for anti-tumor therapy based on the tumor-promoting mechanism of TAMs. Many treatments targeting TAMs have shown good results in preclinical studies (Bhattacharya et al., 2021). Also, the combination of TAMs-targeting with traditional therapies has also achieved good results (Dai et al., 2020b). Currently, therapies targeting TAMs focus on two aspects: clearing TAMs from the TME and prompting reverse polarization of M2 macrophages from TAMs to M1 macrophages (Chen et al., 2018; Pathria et al., 2019; Wang et al., 2021).

### 6.1 Clearing tumor-associated macrophages

The TAMs number is positively correlated with CD8+T cell depletion (La Fleur et al., 2021). Triggering receptor expressed on myeloid cells-2 (TREM2) is a TAMs marker that plays an immunosuppressive role in the TME (Binnewies et al., 2021). In mouse xenograft models of sarcoma, colorectal, and breast cancer, anti-TREM2+ monoclonal antibodies can selectively eliminate TAMs from the TME, increasing the infiltration of CD8+T cells in the TME and its effective function, thus promoting anti-tumor immunity (Molgora et al., 2020). Rodriquez-Garcia et al.demonstrated that M2 macrophages express FRβ receptors. In mouse tumor models of ovarian, colon cancer, and melanoma, CAR-T cells can selectively clear M2 macrophages in the TME by targeting FRβ receptors, leading to the accumulation of pro-inflammatory monocytes and increase of CD8+T cell numbers in the TME. This enhances anti-tumor immunity and delays tumor progression (Rodriguez-Garcia et al., 2021). As demonstrated in the aforementioned studies, targeting TAMs shows great potential as a tumor-treatment strategy, however, further studies are needed in order to precisely access how targeting TAMs may affect OSCC-tumor progression.

### 6.2 Reprogramming tumor-associated macrophages

USP7 is a dehydrogenase, which is highly expressed in M2 macrophages but not in M1 macrophages. Specific silencing of USP7 using siRNA or USP7 inhibitors can activate the P38/MAPK pathway and mediate the transformation of macrophages from M2 to M1, thus, enhancing the proliferation and activity of CD8+T cells. Further studies have shown that inhibition of USP7 can also increase the production of PD-L1 in TME, which results in an effective anti-tumor response (Dai et al., 2020a). MARCO refers to macrophage receptors with collagen structures, which are mainly found in M2 macrophages.La Fleur et al. showed that M2 macrophages in TAMs could be reverse polarized to M1 by targeting MARCO, thereby restoring the activity of CD8+T and NK cells, down-regulating Treg cells, and enhancing the anti-tumor capacity of immune system (La Fleur et al., 2021). Several clinical studies targeting TAMs exist, some of which are listed in Table 2.

**TABLE 2:** Studies targeting TAMs in different tumors.

Strategy	Target	Tumor type	Outcome	References
Clearing TAMs	TREM2	MCA-induced sarcomas	TAMs decreased and CD8+T cell infiltration increased	Molgora et al. (2020)
	CCL5	TNBC	The number of infiltrating tumor cells decreased, and the tumor invasion ability decreased	Frankenberger et al. (2015)
	FRβ	glioma	TAMs are depleted and tumor growth is inhibited	Nagai et al. (2009)
	TIM-3	clear cell renal cell	TAMs decrease and enhance anti-tumor immunity	Komohara et al. (2015)
		carcinomas		
Reprogramming TAMs	CD206	ovarian cancer, melanoma, and glioblastoma	Macrophages repolarized, triggering anti-tumor immunity	Zhang et al. (2019)
	CD206	pancreatic cancer	Macrophages repolarized, Activate adaptive and innate anti-tumor immunity	Jaynes et al. (2020)
	USP-7	lung cancer	M2 macrophages polarize into M1 macrophages	Dai et al. (2020b)
			CD8+T cells increase	
	MARCO	lung cancer	Macrophages polarize into M1 macrophages,CD8+T and NK cell activity increased	Fleur et al. (2020)
	CSF-1/CSF-1R	glioma	the number of TAMs was not decreased, the M2 macrophage markers were decreased, and the tumor-promoting function was impaired	Pyonteck et al. (2013)

## 7 Conclusion

TAMs are important immune cells in the microenvironment of OSCC, and the proportion of M2 macrophages gradually increases with tumor progression. Some studies have shown that the number of TAMs is negatively correlated with the prognosis of OSCC. Many studies have revealed that TAMs promote the invasion, metastasis, angiogenesis, and immunosuppression of OSCC by synthesizing and releasing a variety of growth factors, cytokines, chemokines, and proteolytic enzymes, which ultimately promotes the progression of OSCC. Several reports were made on immunotherapy targeting TAMs on lung, gastric, and breast cancer, whereas few studies have investigated this form of therapy OSCC. Hopefully more studies will reveal the role of TAMs in OSCC, as well as evaluating the consequences of targeting TAMs in OSCC.
